# Isobologram Analysis: A Comprehensive Review of Methodology and Current Research

**DOI:** 10.3389/fphar.2019.01222

**Published:** 2019-10-29

**Authors:** Ruo-yue Huang, Linlin Pei, QuanJin Liu, Shiqi Chen, Haibo Dou, Gang Shu, Zhi-xiang Yuan, Juchun Lin, Guangneng Peng, Wei Zhang, Hualin Fu

**Affiliations:** Department of Pharmacy, College of Veterinary Medicine, Sichuan Agriculture University, Chengdu, China

**Keywords:** drug interaction, synergy, isobologram analysis, principle, application

## Abstract

Drug combination is a common method for clinical disease treatment. Whether the combination of drugs is reasonable often affects the result of the disease treatment. Many methods have been used to evaluate interaction between drugs to date. Isobologram analysis has been mathematically proven and widely used to evaluate drug interactions. In this paper, the principle of isobologram analysis and its application in drug interaction evaluation are summarized. The applications of the similar cotoxicity coefficient and fractional inhibitory concentration index in the evaluation of drug interaction are also reviewed. This work is expected to evaluate the effect of formulations scientifically and provide scientific judgment standards for the development of formulations and clinical drug compatibility.

## Introduction

Drug research and manufacturing versus disease treatment of clinicians is presented as independent actions; the latter necessitates the former, or drug research leads to demand from clinicians, which results in the supply from manufacturers. Generally, for treatment, two or more drugs are clinically used together or successively. The probability of the occurrence of interaction after drug combination increases, which can affect the efficacy and cause safety problems. Generally, the combination of clinical drugs is much more effective than the sum of their individual effects, which is called synergy, and the therapeutic effect is weakened, which is called antagonism. Synergy indicates that two or more components are mixed together, and the effect is greater than the sum of the effects of the individual components when applied alone, thereby producing “1+1 > 2” effect. In the framework of isobolographic analysis, antagonism is higher than the expected doses of drugs A and B that are required to produce the target effect. At present, the clinically combined use of drugs is extremely common because the therapeutic effect of combined drugs is generally better than that of a single drug. For example, the application of pranoprofen and Dianbishu in treating postoperative inflammation in patients with cataract decreases ocular symptoms and sign score and improving macular fovea thickness (Ying-Ying, 2018). Benazepril, combined with amlodipine, can effectively control blood pressure and prevent the occurrence of complications with high safety (Wang and Li, 2018). The intra-articular hyaluronic acid (IA-HA), combined with anti-inflammatory drugs (IA−HA+AI), exhibits considerable efficacy for pain relief compared with IA-HA alone. The analgesic effect of HA in combination with anti-inflammatory drugs (for example, HA combined with doxycycline or with traditional Chinese medicine, such as HA combined with kanggu zengsheng pills) has better effect than HA alone (Lu et al., 2013; Yong and Yanchun, 2015; Euppayo et al., 2017). This phenomenon may come from drug research or a large number of doctors’ reasonably compatible summary of clinical experience. From the clinical efficacy point of view, combined drugs exhibit superiority to single drug, which can increase the efficacy of the drug or reduce the side effects. However, whether two drugs have synergistic effect remains to be proven experimentally.

Given the complexity of clinical manifestations, doctors tend to prescribe drugs in combination. Therefore, the in-depth study of drug interactions can not only provide a theoretical basis for the development of new compound preparation but also provide medication guidelines for clinicians. The combination of the two drugs is more effective than the single drug, and the two drugs are considered to have a synergistic effect. However, this phenomenon is generally not the case. The effect of a combination of two drugs is not necessarily more effective than that of a single drug. Proving whether the interaction produced by drug combination is synergistic is difficult according only to the perspective of curative effect. Therefore, several scientific evaluation methods should be used to evaluate the combined effect of drugs. At present, the existing methods in evaluating compounds commonly include effect surface method, Q value method, ANOVA, equal radiation analysis method, algebraic analysis method, orthogonal analysis method, cluster analysis method, and withdrawal analysis method to evaluate compounds. In this paper, the application of isobologram analysis, cotoxicity coefficient (cotoxicitycottelent [CTC]) partial bacteriostatic concentration, and fractional inhibitory concentration indices (FICIs) are reviewed in the evaluation of drug interactions on the basis of the principles of isobologram analysis.

## Isobologram Analysis

### Principle of Isobologram Analysis

The isobologram analysis that originated from the isobologram is proposed by Loewe in 1953; however, it did not attract attention at that time. The results of Gessner et al. (1970) on the combination of ethanol and chloral hydrate also did not attract people’s attention until the 1970s. From the application of Loewe additivity model in 1992 to the adjustment of ED_50,add_ and their 95% confidence intervals and variances by researchers (Fei and Jisheng, 1998) and to the most commonly used contour graphic analysis method today showed that such method is now widely used in pharmacological experiments and clinical practice.

**Figure 1:**
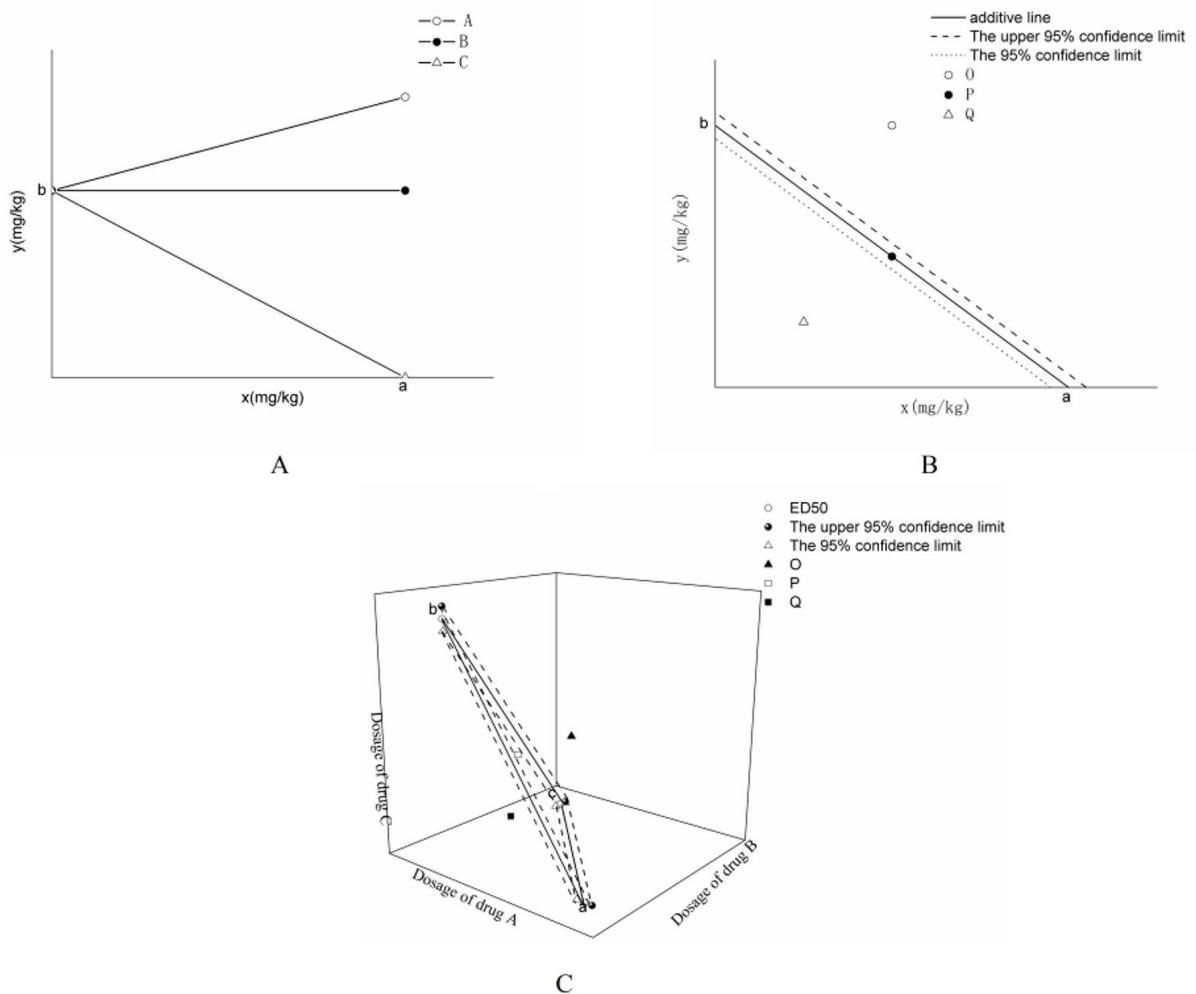
Classification of drug interactions. **(A)** is the classification of drug interactions. **(B)** is the isobologram of the interaction between two drugs. **(C)** is the isobologram of the combination of three drugs in Figure 1.

In general, the efficacy of combining two drugs is shown in **Figure 1A**. The x- and y-axes represent the doses of drugs X and Y, respectively, and a and b represent the dose of the same efficacy when two drugs are used alone, which is generally expressed as half effective dose (i.e., ED50). The plane enclosed by the coordinate system is the equivalent plane, in which all the equivalent points are composed of different doses of drugs X and Y.

If the efficacy of two drugs with different dose compositions is shown in ray A in **Figure 1A**, then the drug Y dose increases with the addition of drug X to produce the same efficacy. Thus, antagonism occurs between drugs X and Y when they are used together. The efficacy agreed with Equation (1), as follows:

(1) $$y=b(1+\frac{x}{k})$$

If the drug effect is shown in the X-ray B (parallel to x-axis) in **Figure 1B**, then the drug Y dose does not change with the addition of drug X when the drug has the same effect. This result indicated that drug X has no effect on drug Y, and the combination of the two drugs is irrelevant.

If the effect produced is shown in line C in **Figure 1A** (the line between points a and b) with the addition of drug X, then the drug Y dose can be lower than the effective dose to produce the same efficacy. When the combination of the interaction between the two drugs is added, it produces an effect that conforms with Equation (2), which is called the addition line, as follows:

(2) $$\frac{x}{a}+\frac{y}{b}=1.$$

The basis for judging drug interactions is to establish the additive line first. As shown in **Figure 1B**, doses a and b producing therapeutic effects when the two drugs are used alone are first obtained, and the a and b phases are connected as an additive line. When two drugs are used in combination to produce the same effect, the horizontal and vertical coordinates are taken as the dose of each drug, and the synergistic effect between the two drugs can be judged according to the position of the corresponding dose on the contour map. If the points actually producing the same efficacy are located above (point O in **Figure 1B**), below (point Q in **Figure 1B**), and above the additive line (point P in **Figure 1B**), then the interactions between the drugs are antagonistic, synergistic, and additive, respectively.

### Test Method in Determining Drug Interaction by Isobologram Analysis

The most classic method in studying drug interactions is the isobologram analysis, which is also known as the contour method. This method has been proven and demonstrated mathematically and used as the basis for the development of most methods that are currently available.

The premise of this method is to satisfy the dose-effect relationship, and the ED50 value is generally calculated using the Dixon-Mood (Yingxiu et al., 2014; Wenkai et al., 2016) and probit methods (Xuejun et al., 2016; Hongxin et al., 2017; Hongwei et al., 2018). The dose-effect relationship function curve between drugs cannot be extremely different. In the isobologram method, the potency ratio is defined as the dose ratio of two drugs when the same drug effect is achieved.

The basic test method involved the following procedures (Jin et al., 2006). First, the dose gradient of drugs A and B (at least four doses) is set, and an experiment is conducted. Next, the ED_50_ value (i.e., 50% efficacy) is found when used alone. In practice, LD_10_ and LD_90_ can also be used (Liu et al., 2017). Then, the two drugs are prepared by using a certain dose gradient (at least four doses) according to the compound ratio and conduct an experiment to determine the ED_50_ measured value (ED_50_ mixture, i.e., the dose of a compound that achieves 50% efficacy) when they are used together.

The potency ratio (*R* *_B_*) of drugs A and B can be calculated using Equation (3), as follows:

(3) $$R_{B}=ED_{50,A}/ED_{50,B}.$$

Then, assuming that the interaction between the two drugs is a simple addition, the ED_50_ theoretical value is calculated when the two drugs are used together by using Equation (4), as follows:

(4) $$ED_{50,\mathrm{add}}=ED_{50,A}/(P_{A}+R_{B}\times P_{B})$$,

where *P* *_A_* and *P* *_B_* are the proportions of drugs A and B in the compound, respectively.

Equation (4) can also be expressed as follows (Equation (5)) (Tabashnik, 1992):

(5) $$ED_{50,\mathrm{add}}={\left(\frac{P_{A}}{ED_{50,A}}+\frac{P_{B}}{ED_{50,B}}\right)}^{-1}$$.

When three drugs are used in combination, Equation (6) can be obtained, as follows:

(6) $$ED_{50,\mathrm{add}}={\left(\frac{P_{A}}{ED_{50,A}}+\frac{P_{B}}{ED_{50,B}}+\frac{P_{C}}{ED_{50,C}}\right)}^{-1}$$.

Therefore, when the plurality of drugs is combined and when a plurality of drugs is used in combination, ED_50_ can be calculated using Equation (7), as follows:

(7) $$ED_{50,\mathrm{add}}=(\sum_{i=1}^{n}\frac{P_{i}}{ED_{50,i}}{)}^{-1}.$$

The equation above can also be expressed as follows:

(8) $$ED_{50,\mathrm{add}}=ED_{50,A}/(P_{A}+\sum_{i=2}^{n}Ri\times Pi)$$,

where n is the number of drug types for combination therapy; *R* *_i_* is the titer ratio between drugs A and I; *P* *_A_* and *P* *_i_* are the proportions of drugs A and I in the compound, respectively; and ED50,i is the ED50 value of drug I when used alone.

*t*-test is generally adopted to carry out statistical analysis on ED_50,add_ and ED_50,mix_ and calculate the 95% confidence interval and variance.

The judgment method of drug interaction is as follows: if ED_50,add_ and ED_50,mix_ are shown at points P, O, and Q, as shown in **Figure 1B**, and if the difference between ED_50,add_ and ED_50,mix_ (ED_50,mix_ is within the 95% confidence interval of the additive line) was insignificant, then two drugs show additive effect. If ED_50,add_ is significantly lower than ED_50,mix_ (ED_50,mix_ is higher than the upper limit of the 95% confidence interval of the additive line), then antagonism occurs. If ED_50,add_ is significantly higher than ED_50,mix_ (ED_50,mix_ is lower than the lower limit of the 95% confidence interval of the additive line), then a synergistic effect occurs between the two drugs.

As shown in **Figure 1B**, the intersection points between the additive line and the x- and y-axes are the ED_50_ values of drugs A and B when used alone, and the dotted lines represent the upper and lower limits of their 95% confidence intervals, respectively.

The traditional isobologram analysis cannot judge the intensity of drug interactions. To evaluate drug interactions more scientifically, we improved the isobologram analysis. This method can be used to judge the strength of drug interactions and evaluate the properties of multiple drug interactions.

The improved isobologram method uses the interaction index (γ) to evaluate drug interactions (Tallarida, 2002); it also uses statistical methods (*t*-test). γ can be calculated using Equation (9), as follows:

(9) $$\gamma =\sum_{i=2}^{n}\frac{d_{i}}{D_{i}},$$

where *Di* represents the dose required by each drug to achieve 50% efficacy when acting alone, and di represents the dose required by each single drug in the compound when the drug combination achieves 50% efficacy. When the interactions between drugs are antagonistic, synergistic, and additive, the corresponding values are >1, < 1, and = 1, respectively; the smaller the value is, the stronger the synergistic effect will be.

The interaction coefficient (R) can also be used to evaluate drug interactions, as follows (Equation (10)):

(10) R=1y.

The larger the R is, the stronger the synergistic effect will. R also requires a one-sample *t*-test.

If three drugs are used together, then the equivalent figure is shown in **Figure 1C**. The figure shows that the plane formed by a, b, and c is the additive surface, and the dotted line is connected by the 95% confidence interval of the ED_50_ value of the three drugs. O, P, and Q are found in the upper, lower, and space of the 95% confidence interval, respectively. The method of judging the interaction of the three drugs is the same as that when two drugs are combined.

## Nonlinear Isobologram Model

**Figure 2:**
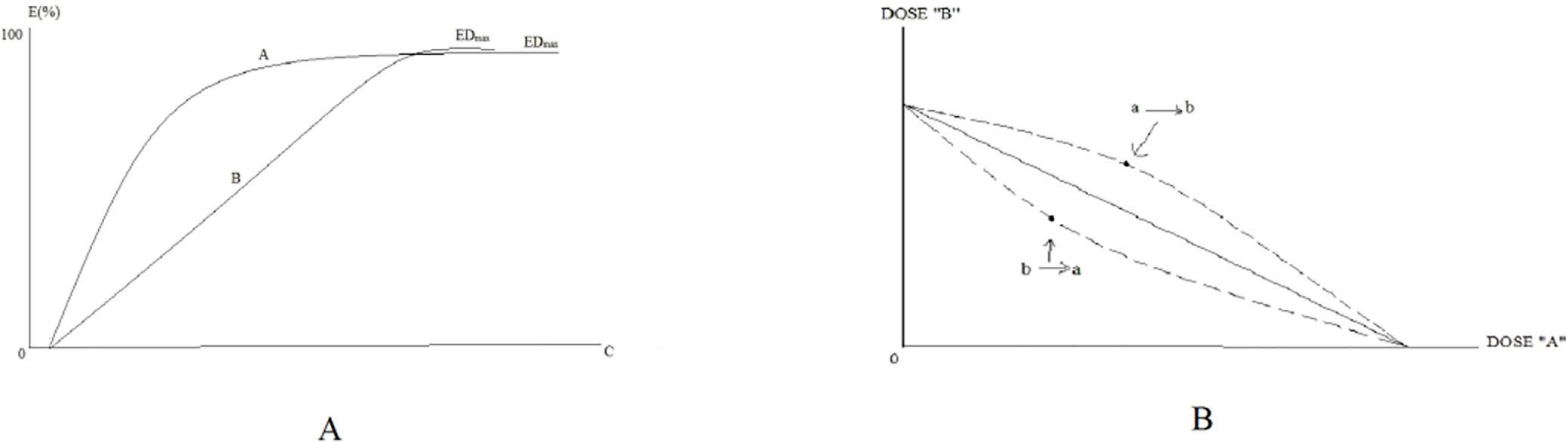
Examples of rectangular hyperbolic dose-effect curves. **(A)** is the example of rectangular hyperbolic dose-effect curves. **(B)** is the isobologram of the interaction between two drugs.

The addition line is nonlinear if the relative potency of the drugs A and B is variable (i.e., the effect curve is shown in **Figure 2A**). When the potency ratio of the two drugs varies, the addition line is not a straight line but a curve, as shown in **Figure 2B**. The dotted lines are equally valid alternate solutions for the isobole for an E_max_ of 50% (yielded by doses A and B) for drugs A and B, with dose-effect curves given by the Hill equation, possessing the same E_max_ but have relative effectiveness, which changes with the effect level. The upper dotted line results from transforming drug A doses to their equivalent doses of drug B, and the lower dotted line results from transforming drug B doses to their equivalent drug A doses.

**Figure 3:**
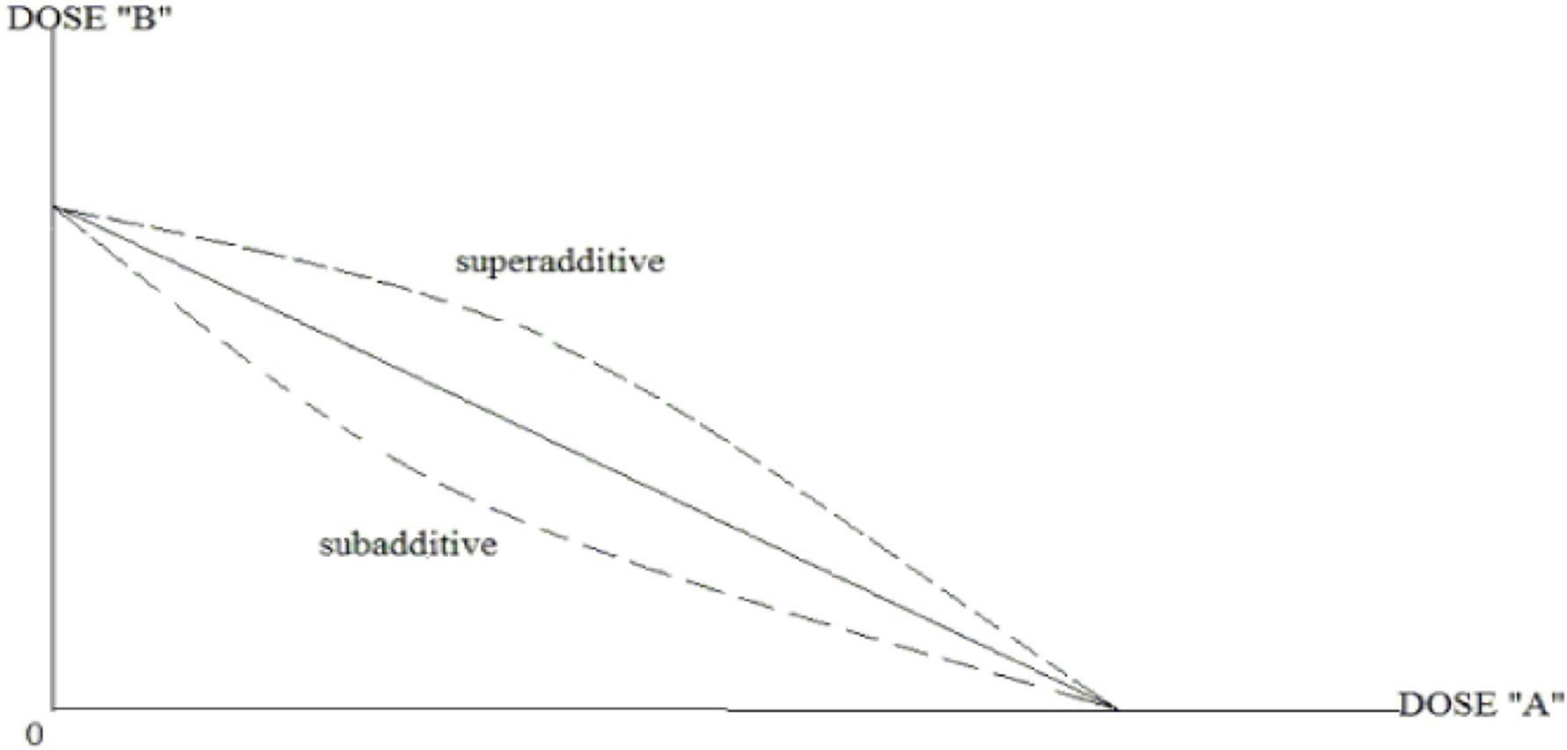
The typical subadditive and superadditive isoboles.

A nonlinear isobologram model can be used to judge whether the effect produced by the combination of two drugs is a synergistic effect. When the combination results in a reduced effect, a considerable quantity of drugs A and B can be needed to achieve the effects, and the point (a, b) will appear above the additive line, which is called superadditive. Conversely, the point (a, b) will appear below the additive line, which is referred to as subadditive. The set of superadditive points demonstrates upward concavity, whereas the subadditive set shows downward concavity. The typical subadditive and superadditive isoboles are shown in **Figure 3**.

Chen and Pounds (1998) proposed a nonlinear isobologram model to analyze the joint action of chemical mixtures for quantitative dose-response relationships, and the model can be used specifically for chemical mixture research.

This nonlinear isobologram model for binary mixtures can be expressed as follows (Chen and Pounds, 1998):

(11) $$\gamma =\gamma_{\min}+\frac{\gamma_{\max}-\gamma_{\min}}{1+e^{-(\beta_{0}+\beta_{1}d_{1}+\beta_{2}d_{2}+\beta_{12}d_{1}d_{2})}}$$,

where γ_min_ is the parameter associated with the minimum response, γ_max_ is the parameter associated with the maximum response, β_0_ is the parameter associated with the effect of the control dose levels, β_1_ is the parameter associated with the effect of the first chemical, β_2_ is the parameter associated with the effect of the second chemical, and β_12_ is the parameter associated with the interaction of the two chemicals.

The mathematical proof of the nonlinear isobole and the situation that leads to it are reported by Grabovsky and Tallarida (Grabovsky and Tallarida, 2004). The mathematical proof of the nonlinear isobole is further described by Tallarida (Tallarida, 2006); the equation is shown as follows:

(12) $$b=E_{i}-\frac{E_{50}}{{\left[\frac{E_{B}}{E_{C}}\left(1+\frac{A_{50}^{q}}{a^{q}}\right)-1\right]}^{1/p}}$$,

where *E* *_B_* is the maximum effect, *E* *_C_* is the maximum effect of the low efficacy drug, A_50_ is the dose of drug A that produces the half of maximum effect *E* *_c_* of drug A, *p* and *q* are the Hill coefficients, *E* *_i_* represents the dose of the individual chemical that produces the specified effect, and a represents the dose required by each single drug in the compound when the drug combination reaches the same effect.

## Application of Isobologram Analysis to Evaluate Drug Interaction

### Application of Isobologram Analysis in Zoopery

The algebraic analysis is evaluated by the R, which is the sum of the ratio of the dose of each drug in the compound to the dose when used alone and when the unilateral and compound produce 50% drug efficacy. The principle and application of the ratio of this analysis is similar to that of isobologram analysis. Using radiation and methods such as algebraic and analytic methods to study the hypnotic effect of rats (Jing, 2007; Jingwen et al., 2007) showed that the synergistic effects of emulsified isoflurane, fentanyl, and midazolam are strong.

The researchers established a nonlinear isobologram and found that the combination of cocaine and cannabinoids produced a strong withdrawal symptoms (Raffa et al., 2007). Detailed isobolographic analysis indicated additivity at EC10 to EC30 and antagonism at low levels (EC-NOEC to EC5) for the compound to 3-benzylidene camphor, benzophenone-1, and benzophenone-2 (Kunz and Karl, 2009). The study used the improved isobologram method to evaluate the effects of the saponins of *Glycyrrhiza uralensis* and *Platycodon grandiflorum* by eliminating phlegm and treating inflammation. The researchers found synergistic or additive effects, which are dose related. When the dosage is within a certain range, the two drugs demonstrate synergistic expectorant effect but only additive effect against inflammation (Bin et al., 2007; Yang, 2006). The combination of methylene blue and tafenoquine had synergistic effect on the CQS strain of *Plasmodium falciparum* (Gorka et al., 2013). Cladribine combined with bendamustine should be used in the treatment of childhood acute lymphoblastic leukemia cells (Goto et al., 2016).

CTC, isobologram, and isobolographic analyses are used in weeding (Gao et al., 2011; Zhao et al., 2018) and memory enhancement (Johnson et al., 2011; Ahmadi-Mahmoodabadi et al., 2016; Nasehi et al., 2017), as well as analgesic (An-Kuo et al., 2018; Basting et al., 2019; Chou et al., 2019; Eiselt et al., 2019; JI et al., 2017; Li et al., 2019), antioxidant (JI et al., 2017; Yao et al., 2017), anti-inflammation (Rakariyatham et al., 2018), anticancer property examination (Chao et al., 2019; Şiğva et al., 2019), cytotoxicity (Tatay et al., 2014; Sir et al., 2018; Hoover et al., 2019), and thyroid examination (Nilufer Ozdemir Kutbay et al., 2017). The analytical methods and other methods used to evaluate drug interactions are shown in **Table 1**.

**Table 1:** Examples of evaluating drug interactions.

Methods	Study type	Therapeuticactivity	Evidence of synergy	References
Cotoxicity Cottelent	*In vitro*	Herbicide	The synergistic action of atrazine was achieved when combined with nicosulfuron at a ratio of 17:3 for crabgrass (*Digitaria ciliaris*) and purslane (CTC=171.9).	Gao et al., 2011
Combination index	*In vitro*	Herbicide	The synergistic herbicidal effect of the butachlor was achieved when combined with halosulfuron-methyl and mesotrione for the microalga *Selenastrum capricornutum* (CI<1).	Zhao et al., 2018
	*In vitro*	Anticancer	The researchers testified to the synergistic effect of paclitaxel with endemic plant extracts (combination index, CI; ED_50_<0.41). The combinations, which indicated the synergistic effect, increased the Bax/Bcl-2 ratio by suppressing Bcl-2 gene expression into the prostate cancer cell lines.	Şiğva et al., 2019
	*In vitro*	Cytotoxicity	The low concentrations of the composition produce additive effects, and the high concentrations produce synergistic effects. The CI values for tertiary combinations (zearalenone+α-zearalenol+β-zearalenol) ranged from 2.95 ± 0.75 to 0.41 ± 0.23)	Tatay et al., 2014
Isobologram	*In vivo*	Memory retention deficit	Isobologram analysis showed a synergistic effect between ACPA and m-CPBG on memory consolidation deficit. ED_50,mix_ was significantly lower than ED_50,add_.	Ahmadi-Mahmoodabadi et al., 2016
	*In vivo*	Memory consolidation impairment	Isobologram analysis showed that a synergistic effect between D-AP5 and AM630 on memory consolidation deficit. The difference between ED_50,add_ and ED_50,mix_ was insignificant.	Nasehi et al., 2017
	*In vivo*	Anti-inflammation	Isobologram analysis confirmed that the combined treatments produced a synergy.	Rakariyatham et al., 2018
	*In vivo*	Antinociceptive	Isobologram demonstrated that the synergistic response in antinociceptive interaction between *Pterodon pubescens* fruit extract and *Cordia verbenacea* essential oil in the abdominal writhing test induced by acetic acid. The concave slope of the isobologram plots indicated synergistic interactions between the extracts.	Basting et al., 2019
	*In vivo*	Antinociceptive	Isobologram demonstrated that serotonin produces a synergistic antinociceptive interaction with oxybuprocaine or proxymetacaine. ED_50,mix_ was significantly lower than ED_50,add_.	Chou et al., 2019
	*In vivo*	Antinociceptive	Isobologram analyses revealed that the coinjection of An2-NT(8-13) with morphine induced an additive analgesic response. Every combination felt on the theoretical line of additivity for these drugs.	Chou et al., 2019
	*In vivo*	Producing cutaneous antinociceptive effects	A significant depth of the sensory block with bupivacaine+serotonin in producing cutaneous antinociceptive effects was also found. ED_50,mix_ was significantly lower than ED_50,add_.	JI et al., 2017
	*In vivo*	Skin antinociception	Epinephrine synergistically enhanced the effects of dibucaine, whereas phentolamine partially blocked these effects.	An-Kuo et al., 2018
	*In vivo*	Skin antinociception	The synergistic effect was achieved on prolonged antinociceptive duration when dopamine is combined with dextrorphan. ED_50,mix_ was significantly lower than ED_50,add_.	Li et al., 2019
	*In vitro*	Anticancer	The isobol curve and CI plot revealed a typical synergistic interaction between DET and CP with the CI of <1, thereby suggesting that DET sensitized CP in inhibiting melanoma cells.	Chao et al., 2019
	*In vitro*	Cytotoxicity	The mixtures of PFOS and PFHxS were still considered additive. The values for mixtures of PFOS and PFHxS approximate mode II(A) isobole line.	Hoover et al., 2019
	*In vitro*	Cytotoxicity	The combination of tacrolimus and oxytocin was accepted as antagonistic on ADMSC. The CI value of the FK506 and OT combination was 1.24.	Sir et al., 2018
	*In vitro*	Antithyroid cancer cell	The combination of metformin and pioglitazone was determined as additive.	Nilufer Ozdemir Kutbay et al., 2017
Isobologram and Interaction index	*In vitro*	Antioxidant	The synergistic antioxidant effect was achieved when tea extract is combined with ascorbic acid. ED_50,mix_ was significantly lower than ED_50,add_.	Enko and Gliszczyn´ska-S´wigło 2015
Isobolographic analysis	*In vitro*	Antioxidant	The highest synergistic antioxidant effect of the strawberry extract was achieved when combined with the mango extract at the ratios of 1:9 and 1:1 (V/V) in scavenging DPPH and ABTS radicals, respectively. ED_50,mix_ is significantly lower thanED_50,add_.	Yao et al., 2017
	*In vivo*	Short-term learning and memory	The superadditive combination was 5-HT 2+/5-HT 7- for short-term learning and memory. Experimental drug combination (IC_50_ values) was above the theoretical IC_50_ isobole.	Johnson et al., 2011

### Application of Isobologram in Evaluating Synergistic Effect of Bacterial Growth Inhibitory

For microorganisms, such as bacteria and fungi, the complexity of infection often involves the combined drug use. In pharmacology, *in vitro* combined drug susceptibility tests are generally performed to determine whether the two drugs have a synergistic effect. FICIs are often used to judge drug interactions.

Generally, the method introduced by the American Committee for Clinical Laboratory Standardization (CLSI) is referred to for experiments (Fothergill, 2015). The judgment of the minimum inhibitory concentration (MIC) CLSI standard reference, which is typically used to trace the microbroth dilution method and determine the MIC of each drug alone or in combination, and FIC is calculated (Menglan et al., 2018). FIC is similar to the formula for γ of the modified isobologram method. γ is the sum of the ratio of the dose of each drug in the compound to the dose when used alone and when the unilateral and compound produce 50% efficacy. The FICI is the sum of the MIC of the antibacterial drug instead of the ED50. The ratio of the MIC value of each drug in the compound to the MIC value of each drug alone is used to determine the synergistic effect of drugs, which is similar to the isobologram method.

FICI has many criteria, and the following are generally adopted: when the FICI is ≤0.5, >0.5 and ≤1, >1 and ≤2, and >2, the interactions among drugs are synergistic, additive, unrelated, and antagonistic, respectively. The remaining judgment criteria are as follows: when the FICI is ≤0.5, >0.5 and <4, and ≥4, synergy, independence, and antagonism interactions occur, respectively (Stein et al., 2016). When the FICI is >1, =1, >1 and ≤2, and >2, synergy, addition, independence, and antagonism occur, respectively (Fratini et al., 2017). Interpretations often vary.

The results of the *Staphylococcus aureus* bacteriostatic assay (FICI between 1.00 and 1.25) indicated an additive effect of daptomycin and rifampicin (Stein et al., 2016). For *Staphylococcal* infections, *Origanum vulgare* (oregano) and *Leptospermum scoparium* (manuka) essential oils can act as effective alternatives to chemotherapy for staphylococcal infections and increase food safety (Fratini et al., 2017). For methicillin-resistant *S. aureus*, the synergistic effect is good when vancomycin is compatible with L-arginine and ceftriaxone sodium (Chaudhary et al., 2013), lipofectin prepared by gentamicin and piperine (Khameneh et al., 2015), active constituents of *Duabanga grandiflora* inhibited by penicillin-binding protein 2a (PBP2a) and ampicillin (Santiago et al., 2015), β-lapach combined with naphthoquinone antibiotics (FICI between 0.07 and 0.50 (Macedo et al., 2013)), gentamicin and piperine prepared lipid plastid (FICI = 0.5; (Khameneh et al., 2015)), and camel lactoferrin combined with oxacillin or vancomycin (FICI = 0.5, 0.37; (Redwan et al., 2016)). Quercetin combined with some polyphenolic acids (e.g., gallic acid, cinnamic acid) has a synergistic antibacterial effect against *Aeromonas aeruginosa* and has an additive effect on *Edwardsiella tarda* and *Aeromonas hydrophila* (Prasad et al., 2014). For *Escherichia coli*, the synergistic antibacterial effect of kanamycin combines well with metronidazole (Olajuyigbe and Afolayan, 2015), ciprofloxacin, and the alkaloid extract of *Sophora alopecuroides* (Jaktaji and Mohammadi, 2018). The combination of *Berberis aristata*, colistin, and tigecycline has a synergistic effect on carbapenem-resistant *E. coli* (Thakur et al., 2016). For clinically separated *Acinetobacter baumannii*, cefoperazone, tigecycline, and sulbactam in combination with 56.8% and 50.0% FIC of ≤0.5, with 61.4% imipenem combined with sulbactam and the FIC of <1 are observed in the drug (Menglan et al., 2018). Therefore, *A. baumannii* (carbapenem sensitive and carbapenem resistant) can be treated with tigecycline, imipenem, and sulbactam, and sulbactam and cefoperazone. 2-(2-Nitrovinyl) furan combined with amoxicillin (FICI = 0.125) has good synergistic antibacterial effects (Ajiboye, 2018). Cefuroxime combined with levofloxacin has a synergistic effect on *E. faecalis* isolated from endophthalmitis cases (FIC = 0.487) (Suzuki et al., 2017). For *Pseudomonas aeruginosa*, with ceftazidime injection combined with Tanreqing injection and Xiyanping injection, the FIC values are 0.375 and 0.625, with synergistic and additive antibacterial effects, respectively (Penglei and Shaoping, 2018). The study also suggested that the DDTC byproduct of DSF metabolism potentiates the antibacterial activity of DSF for *S. aureus* and *S. epidermidis* as a standalone and combination agent (Frazier et al., 2019).

### Application of Isobologram in Insecticide Evaluation *In Vitro*

*In vitro* antiparasitic drugs or agricultural insecticides are often used in combination to improve insecticidal effects, and the scientific evaluation of the synergistic effect of insecticides is essential. Sun Yunpei proposed the use of CTC to evaluate the insecticidal effect of pesticide mixture. CTC is generally determined by first obtaining the LD50 of each single drug and the compound drug to the parasite and then taking the ratio of the actual toxicity index (ATI) of the compound drug to the theoretical toxicity index (TTI). During the test, the toxicity index (TI) of each insecticide is generally calculated, and TIA = 100, assuming that insecticide A is the calibrator. When three doses are used together, the TI values of pesticides B and C are shown in Equations (11) and (12), as follows:

(13) TIB=LD50,ALD50,B×100,

(14) TIC=LD50,ALD50,C×100.

The ATI of ITO (M) was calculated using Equation (13), as follows:

(15) ATI=LD50,ALD50,M×100.

The TTI of the mixture (M) was calculated using Equation (14), as follows:

(16) $$\mathrm{TTI}=TI_{A}\times P_{A}+TI_{B}\times P_{B}$$.

Finally, the CTC was calculated as follow (Equation (15)):

(17) CTC=ATITTI×100.

After formula transformation, Equation (16) can be obtained as follows:

(18) $$\mathrm{CTC}=\frac{LD_{50,A}}{LD_{50,M}}\times {\left(P_{A}+\frac{P_{B}\times LD_{50,A}}{LD_{50,B}}++\frac{P_{C}\times LD_{50,A}}{LD_{50,C}}\right)}^{-1}\times 100.$$

The following criteria are generally adopted for CTC to judge the compatibility effect of drugs or pesticides: when the CTC values are >120, <80, and between 80 and 120, the synergistic use of pesticides produces synergistic, antagonistic, and additive effects, respectively (Sun and Johnson, 1960). Many criteria for judgment are also present. For example, the CTC indices of >200, <200 and >150, <150 and >70, and <70 indicated evident synergism, partial synergism, addition, and antagonism, respectively (Qingzhen et al., 1993). The CTC values of significantly higher than 100, close to 100, and significantly less than 100 represent synergistic, additive, and antagonistic effects, respectively (Zhongyan et al., 2016).

If the ratio of ED_50,add_ and ED_50,mix_ in the isobologram method is set as X, then the calculation method of X is as shown in Equation (17), which is multiplied by 100; this formula is similar to the formula of the CTC. Therefore, the use of the cotoxicity method is also an application example of isobologram in the evaluation of pesticide mixture.

(19) $$X=\frac{ED_{50,A}}{ED_{50,\mathrm{mix}}}\times {\left(P_{A}+\frac{P_{B}\times ED_{50,A}}{ED_{50,B}}++\frac{P_{C}\times ED_{50,A}}{ED_{50,C}}\right)}^{-1}$$.

The nonlinear isobologram demonstrated that the combination of cocaine and cannabinoid has synergistic effects on planarians (Raffa et al., 2007). When the ratio of rotenone to *Cinnamomum cassia Presl oil* is 1:35, the synergistic effect of rotenone on the third instar larvae is good for *Spodoptera litura* (Li et al., 2017; Wen et al., 2013). Carvacrol combined with thymol has a synergistic effect on *Culex pipiens pallens* (Diptera: Culicidae) and has the potential to be developed as a natural fumigant for mosquitoes (Ma et al., 2014). The combination of *Solanum xanthocarpum* and *Withania somnifera* has synergistic effects on the larvae of mosquito vectors, such as *Anopheles stephenii* (Bansal et al., 2015). For *Pieris rapae*, a synergistic effect on insecticidal activity is observed when the ratio of rotenone to ZnO nanoparticle is 4:1 (Chen et al., 2016). When the ratio of bisdemethoxycurcumin to scopoletin is 6:7, strong acaricidal activity against *Tetranychus cinnabarinus* (Boisduval) (Acari: Tetranychidae) is observed at different developmental stages (Zhang et al., 2016). The combination of three neonicotinoids (thiametholin, thiamethamine, and furfuran) has synergistic toxicity for bees (Liu et al., 2017), and no synergistic effect was observed among clothianidin, propiconazole, and Cr(III) (Sgolastra et al., 2017). Yiming et al. (2017) calculated the combination of β-cypermethrin and avermectins through CTC and good synergistic effects on the production of larvae of *Evergestis extimalis* Scopoli. For Solenopsis invicta (Hymenoptera: Formicidae), the synergistic effect of 2:8 thiacloprid and *Beauveria bassiana* is good (Li et al., 2018).

Isobolographic analysis, which is a graphic method often used in pharmacology to analyze mixture effects, is used to examine the LD_50_ values for the blends visually (Berenbaum, 1989; Greco et al., 1995). The application of isobolographic analysis is the same as that of isobologram, that is, based on whether ED_50,add_ and ED_50,mix_ have significant difference to determine whether the drugs have synergistic effects. Studies have shown that the 50:50 and 20:80 (oil+ethanol) blends exhibit synergy (Sims and O’Brien, 2011). Sun Zhen et al. used the isobolographic analysis to determine that the ratio of *Pulsatilla chinensis saponins* B9 and B7 is 1.5:1, which has a synergistic effect on *Schistosoma japonicum* killing (Zhen et al., 2014). The results showed that the combination of emulsified isoflurane and fentanyl can induce a synergistic effect that can become stronger when the two are combined with midazolam.

Combination index (CI) is used to determine the degree of drug interaction, and its formula is the sum of the ratio of the dose of each drug in the compound to the dose when used alone when the combination and compound produce 50% efficacy. The principle and application are the same as that in isobologram. In the evaluation of earthworm *Eisenia fetida* mortality with this index, the synergistic effect of atrazine with λ-cyhalothrin is good (Chen et al., 2014b), and no synergy was observed between butachlor, imidacloprid, and chlorpyrifos (Chen et al., 2014a). This result indicated the slight synergistic effect of λ-cyhalothrin with Cd (Wang et al., 2015).

### Application of Isobologram in GABA-A Receptor Field

Isobolographic analysis has been used extensively in the GABA-A receptor field, particularly with regard to the synergy of sedative drugs. For instance, the combination of morphine and GABA antagonists (bicuculline or 5-aminovaleric acid) has synergistic effect for antispasmodic (Seiya et al., 2002). When the triazolam-to-pregnanolone ratio is 1:30, the synergistic effect of attenuate rates of food-maintained responding (Gunter et al., 2015). The isobologram analysis showed that an additive effect but not synergistic effect between muscimol and (+)-MK-801 on memory retention deficits in the BLA (Khakpoor et al., 2016). Phenobarbital, as active modulators of GABA A receptor, showed additive interactions when it is combined with oxcarbazepine and zonisamide (Matsumura and Nakaki, 2014). Butane and pentane showed synergistic anesthetic effects *in vivo*, which is consistent with their different *in vitro* receptor effects (Brosnan et al., 2017). Researchers showed that the anticipation become less synergistic as the constitutive activity increases (Germann et al., 2018).

For antiepileptics drugs, the researchers corrected the variance of ED_50,add_, and the results showed that the efficacy of oxcarbazepine combined with phenytoin has additive effects when the ratios are 1:3 and 3:1 or antagonistic effects when the ratio is 1:1 (Luszczki et al., 2003a). When the ratio of tiagabine to gabapentin are three fixed ratios (i.e., 1:3, 1:1, and 3:1), it has synergistic antiepileptic activity (Luszczki et al., 2003b). Joint treatment with DZP and MK-801 as a 3:1 fixed ratio displays synergistic protection (Shakarjian et al., 2015). This result suggested strong positive cooperativity between the combination of diazepam+ketamine+valproate in reducing EEG power and stopping seizure activity (Jerome et al., 2017).

## Discussion

In using isobologram analysis to judge drug interactions, several conditions need to be satisfied. The first condition is that the dose curve of the two drugs should be parallel dose-response curves. Then, two drugs should have the same maximum effects, as shown by the slopes of the curves. For example, the dose curve of the two drugs can be an S-shaped curve or a parabola; thus, their ED_50_ can be calculated. Next, the potency ratio should be constant. Isobologram is limited when both drugs are full agonists with a variable potency ratio. A full and partial agonist can produce a consistent generalization that leads to a single (curved) isobole of additivity (Tallarida, 2006). It is always linear when the individual dose-effect curves have constant potency ratio and curved when such ratio is variable (Tallarida, 2012; Tallarida, 2016). It is valid only for dose-effect curves for which the principles of dose equivalence and sham combination lead to Equation 2, which is the linear Loewe additivity for predicted additivity (Geary, 2013). The author suggested that the criteria in judging synergy metrics, ① face validity, ② mathematical soundness, and ③ utility, with respect to further basic and clinical research are needed.

The last prerequisite is the slopes of the dose-response curves of not equal to 1. The dose curve cannot be a U-shaped curve.

The research has summarized two limitations of isobologram, as follows: one is the fact that Loewe additivity model becomes unusable when a dose-effect curve is unavailable or difficult to model and the fact that the potency ratio is often not constant (Foucquier and Guedj, 2015). The advantage of isobologram is that it enables the complementation of the algebraic analysis with an intuitive, flexible, and widely accepted graphical approach known as isobologram analysis. Isobologram method can intuitively judge the synergistic effect produced by drug combination, which has a perfect mathematical demonstration system and a strict judgment standard. It converts pharmacological problems into mathematical problems and performs calculations, and it provides the possibility to find the real meaning of “collaborative” combination.

The formula for CTC is similar to isobologram analysis, whereas the formulas for FICI, CI, and algebraic analysis are similar to that of γ. Therefore, formula for CTC can be considered from the formulas of FICI, CI, algebraic analysis, and CTC that these methods are an application of isobologram analysis. The advantage of CTC, FICI, CI, and algebraic method is that they can intuitively compare the size of the synergy. The disadvantages and prerequisites of CTC, FICI, CI, and algebraic method may be the same as that of isobologram analysis. However, these preconditions have minimal emphasis to evaluate the synergistic effects of bacterial growth inhibitory previously.

Tim Holland-Letz et al. (2018) showed that D-optimal and combination designs showed to be robust toward wrong assumptions with regard to the kind of interaction expected (Holland-Letz et al., 2018). The more complex an interaction is expected, the more different the mixture ratios should be used. A 3D response-surface analysis spanning the explored region of doses can provide a complete description of the combination effect (Foucquier and Guedj, 2015).

The research has summarized the methods used in judging the synergy of traditional Chinese herbal medicine, including CI, isobologram, and γ (Xian et al., 2016). Chen Cong-cong et al. (2019) also discussed the method of selecting the optimal ratio of traditional Chinese medicine prescription (Cong-cong et al., 2019).

The principles of these calculation methods are the same as that of the isobologram method through formula transformation and may be extended based on the isobologram method. However, the judgment criteria are different from the isobologram method. Although differences exist in the judgment criteria, essentially, every judgment criterion is divided according to the interval, which indicates that only when the range of confidence interval is exceeded, the interaction between drugs changes. This occurrence is caused by the difference between experimental individuals and the error in the experiment. Deviations are also observed in the judgment criteria, which also have an impact on the experimental results. Therefore, the accurate selection of judgment criteria is the key to the evaluation of the combined effects of drugs.

The premise of these methods (i.e., CTC, FICI, isobologram, and so on) is to satisfy the dose-effect relationship. However, in actual experiments, the calculation of the dose-effect relationship is occasionally impossible, and only the time-dependent relationship can be judged. In the isobologram method, the potency ratio is the dose ratio of the two drugs when the same drug effect is achieved. Therefore, ED50 or LD50 may be replaced by LT50, and the improved isobologram method or CTC can be used to judge drug interaction. However, this idea has yet to be verified.

## Author Contributions

R-yH collected references and wrote the main part of the manuscript. LP collected references and made suggestions. QL, SC and HD collected references. GS, Z-xY, GP, WZ and JL made suggestions. HF participated in the coordination of the study and reviewed the manuscript. All authors read and approved the final manuscript.

## Conflict of Interest

The authors declare that the research was conducted in the absence of any commercial or financial relationships that could be construed as a potential conflict of interest.
